# Supplementary figures and images for: A quantitative genetic model of background selection in humans
Source: PLoS Genet. 2024 Mar 20;20(3):e1011144. doi: 10.1371/journal.pgen.1011144 (PMC10984650; doi:10.1371/journal.pgen.1011144)

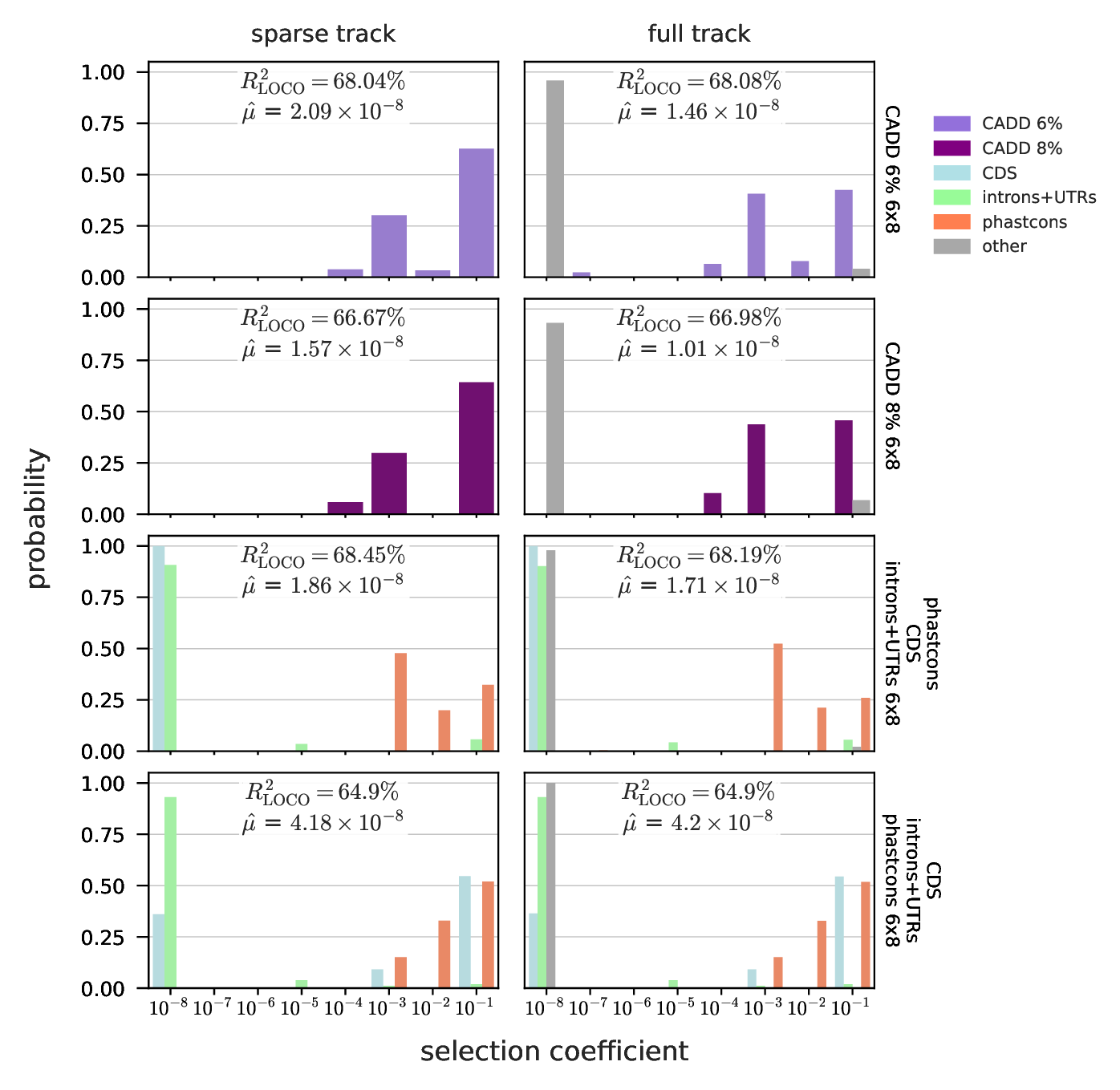

Supplement: S1 Fig — (TIF) [file pgen.1011144.s002.tif]

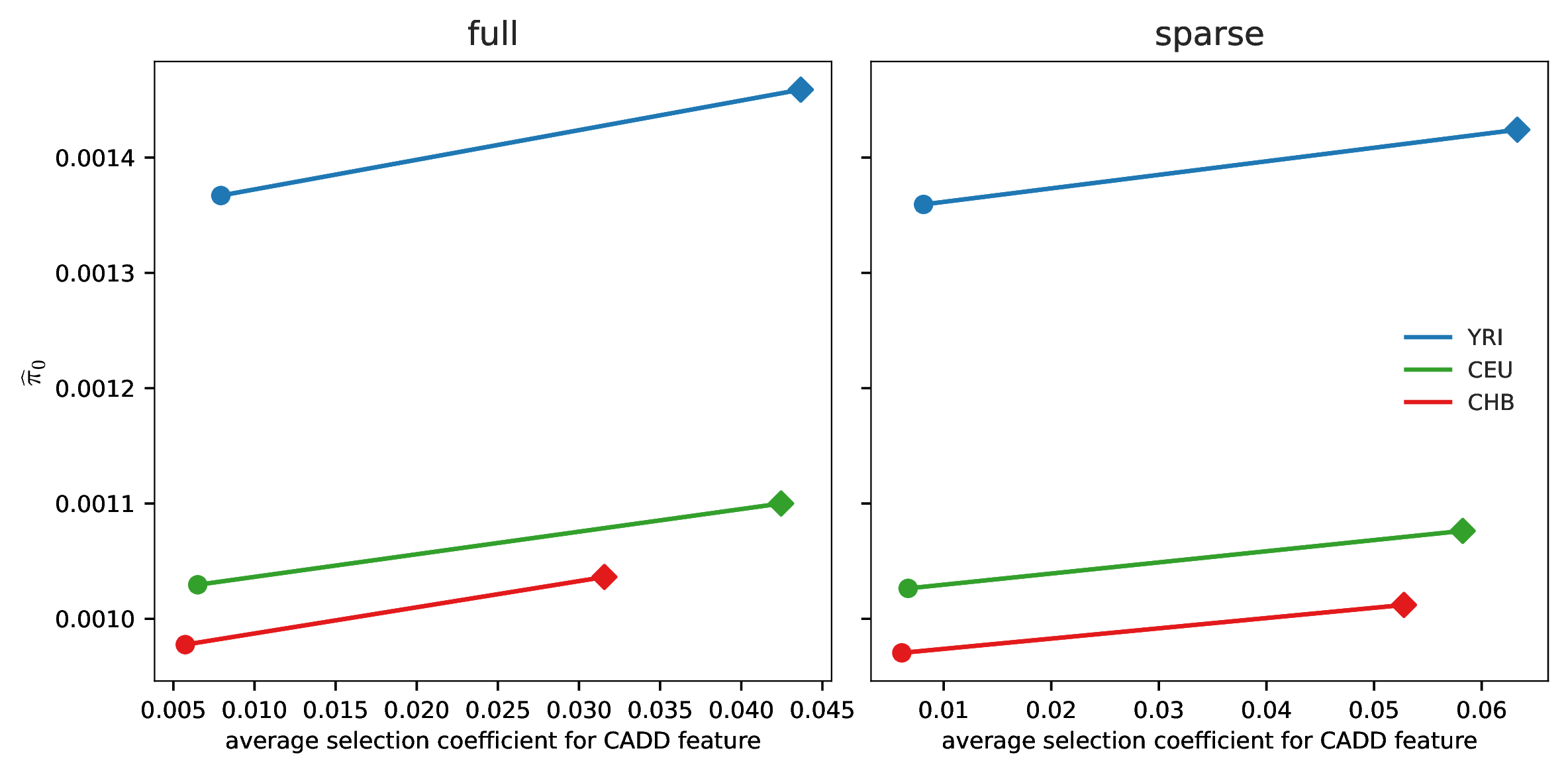

Supplement: S2 Fig — Diamonds indicate estimates under the strong selection grid (up to s = 10−1) and circles indicate estimates under the default grid (up to s = 10−2). (TIF) [file pgen.1011144.s003.tif]

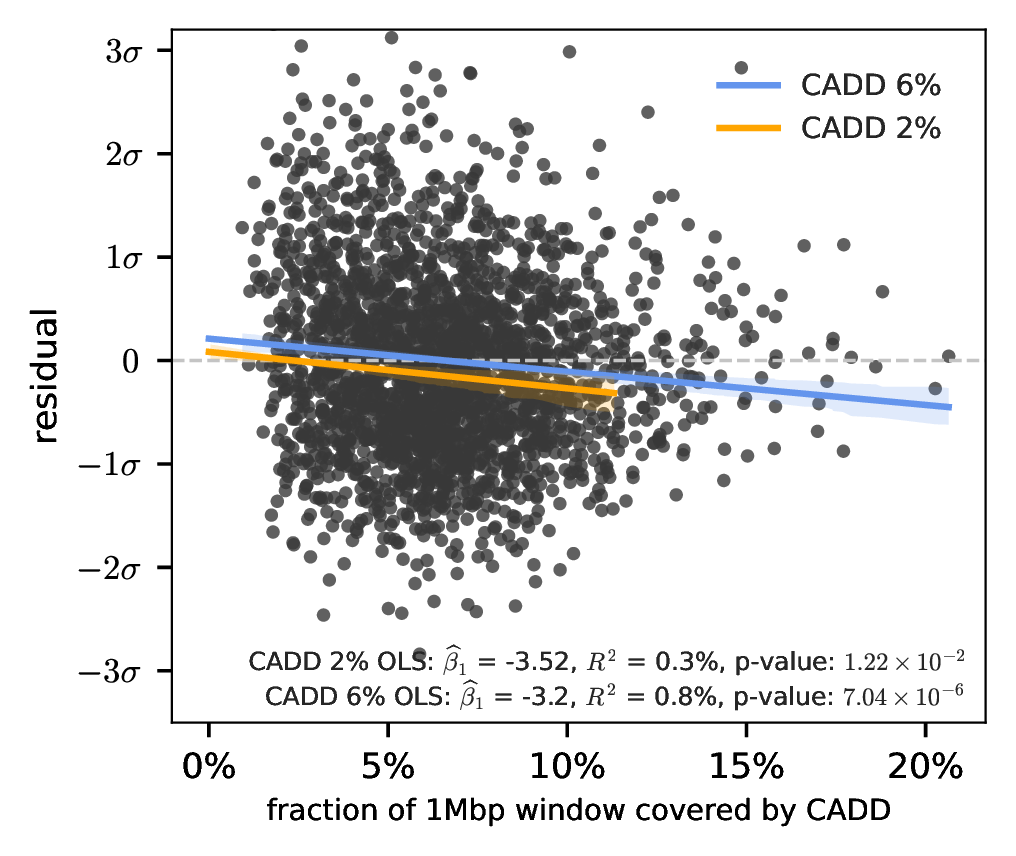

Supplement: S3 Fig — (TIF) [file pgen.1011144.s004.tif]

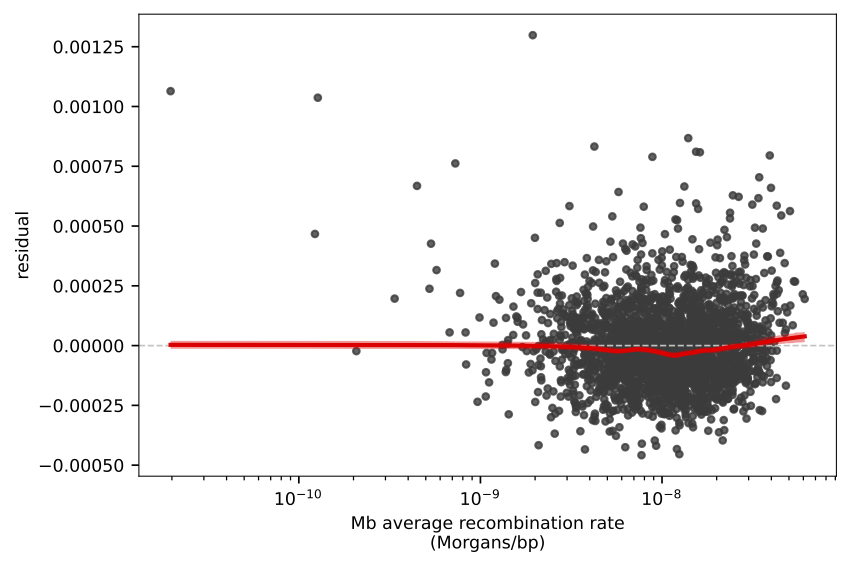

Supplement: S4 Fig — (TIF) [file pgen.1011144.s005.tif]

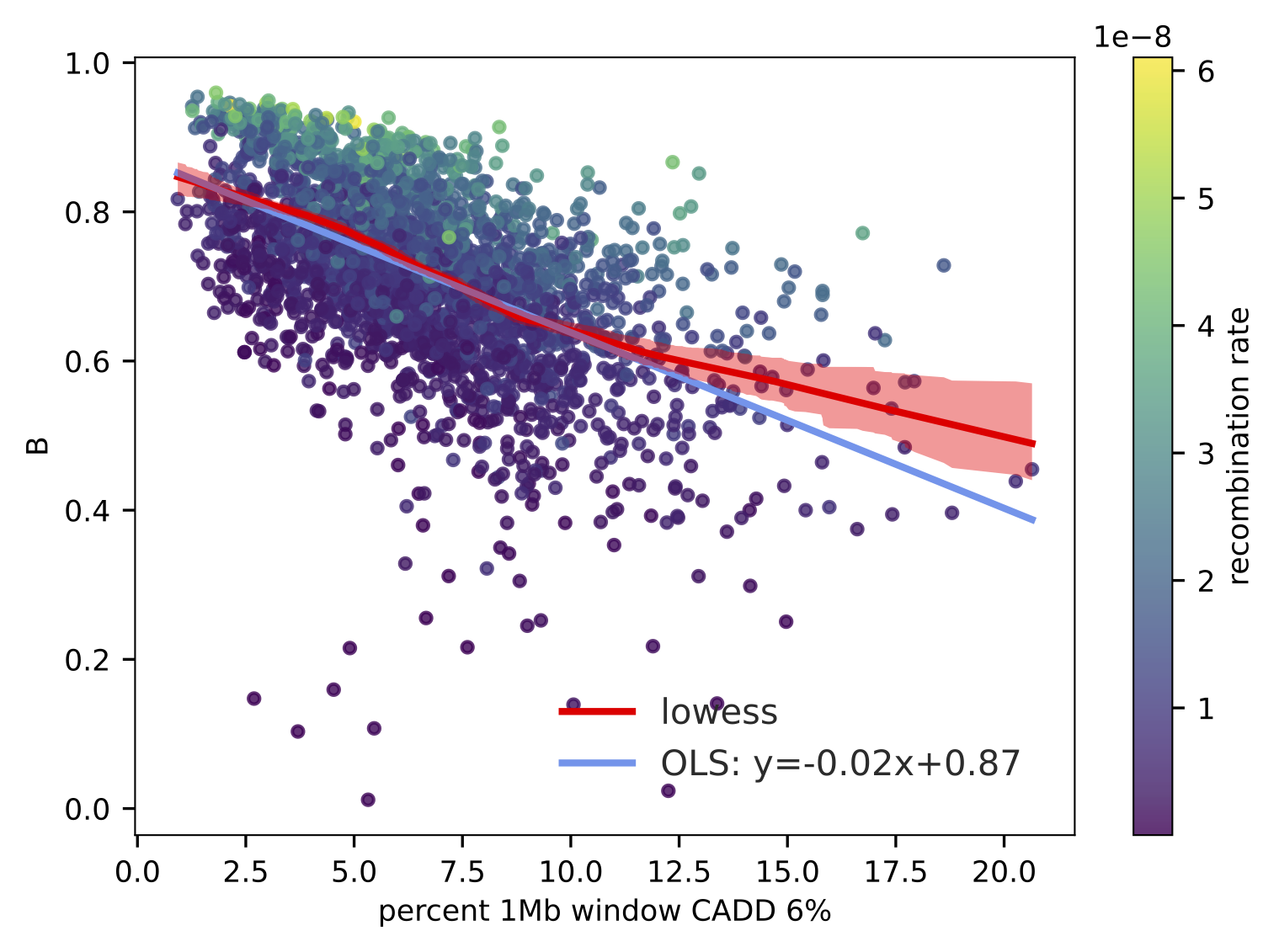

Supplement: S5 Fig — (TIF) [file pgen.1011144.s006.tif]

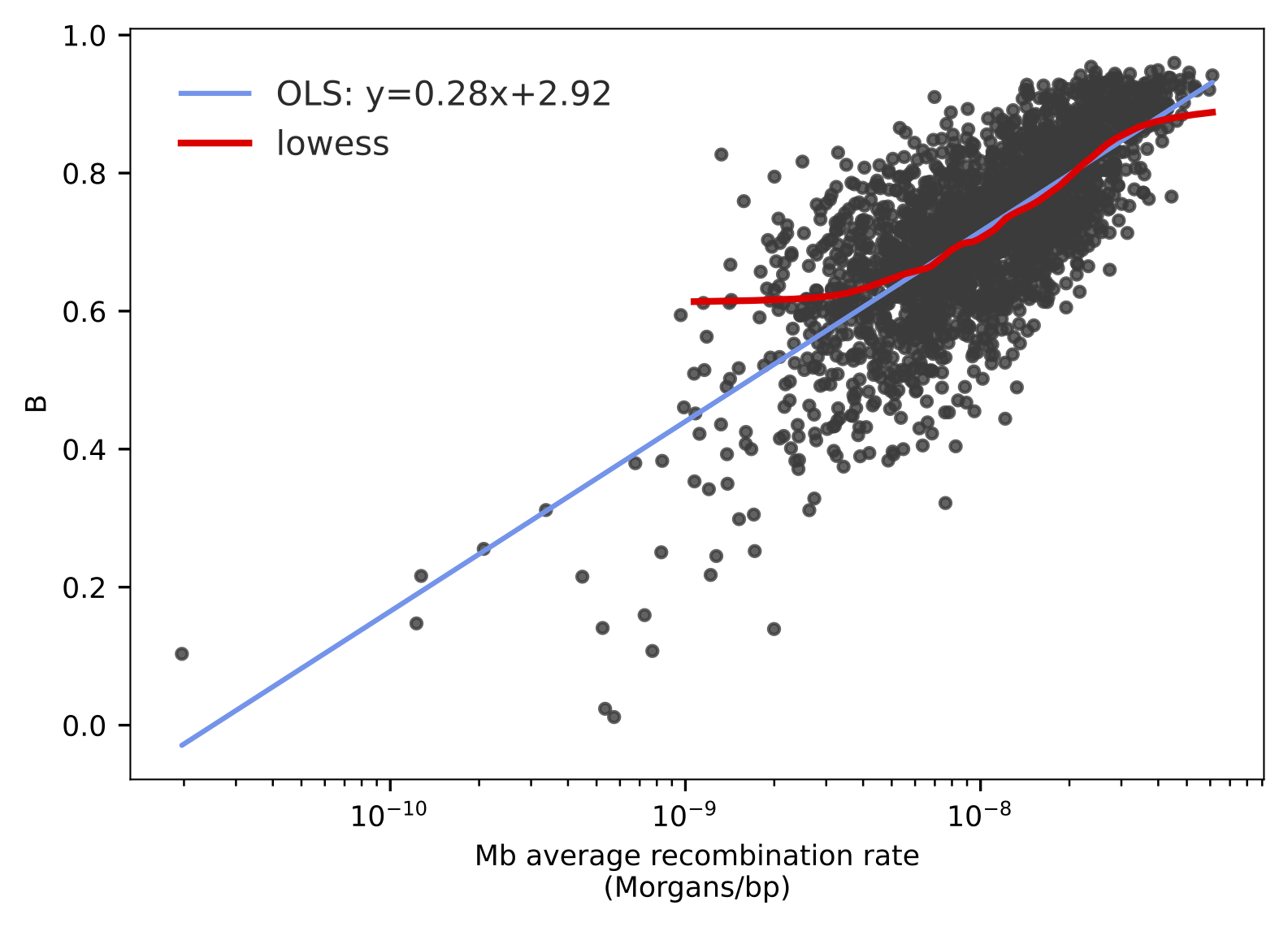

Supplement: S6 Fig — (TIF) [file pgen.1011144.s007.tif]

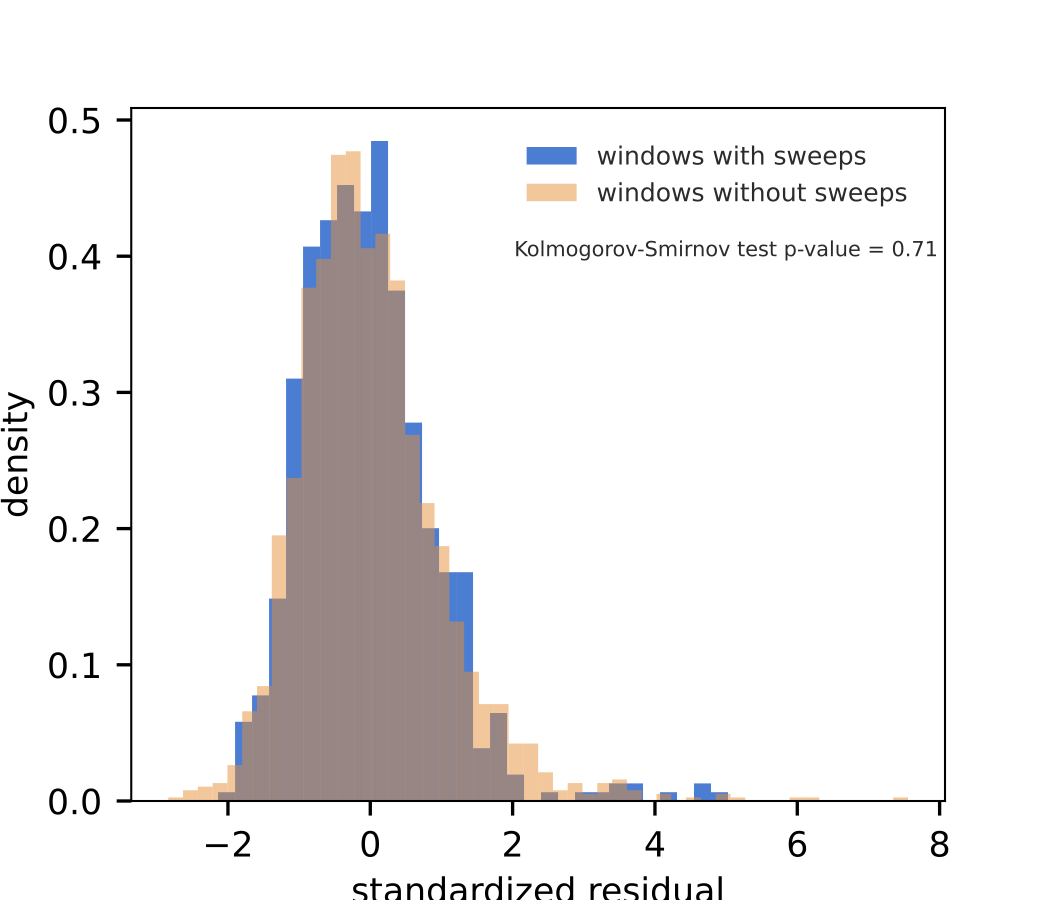

Supplement: S7 Fig — (TIF) [file pgen.1011144.s008.tif]

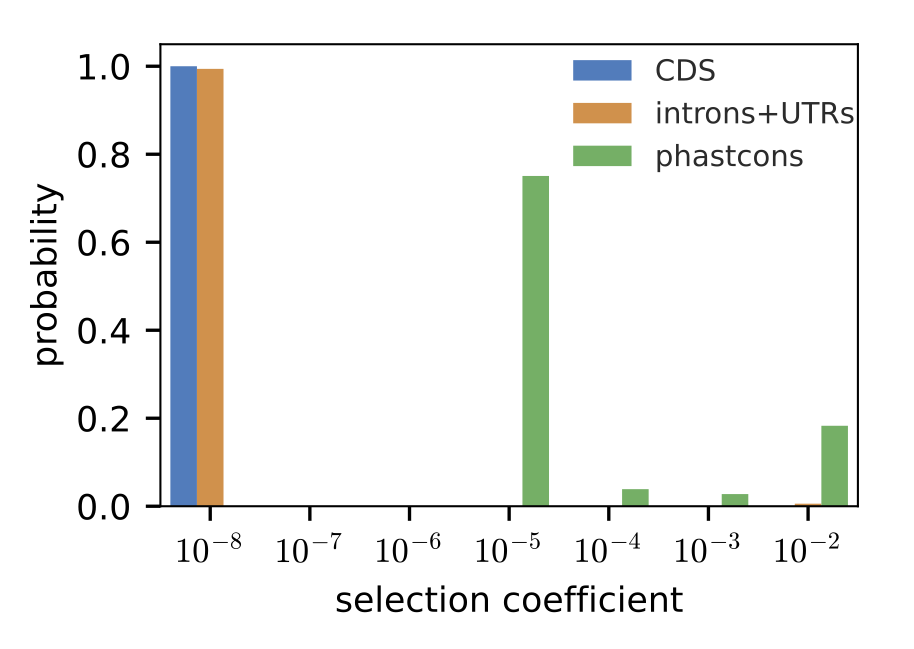

Supplement: S8 Fig — The maximum likelihood mutation rate estimate for this is μ^=8×10^-8^, which is the upper boundary of the range used during optimization. (TIF) [file pgen.1011144.s009.tif]

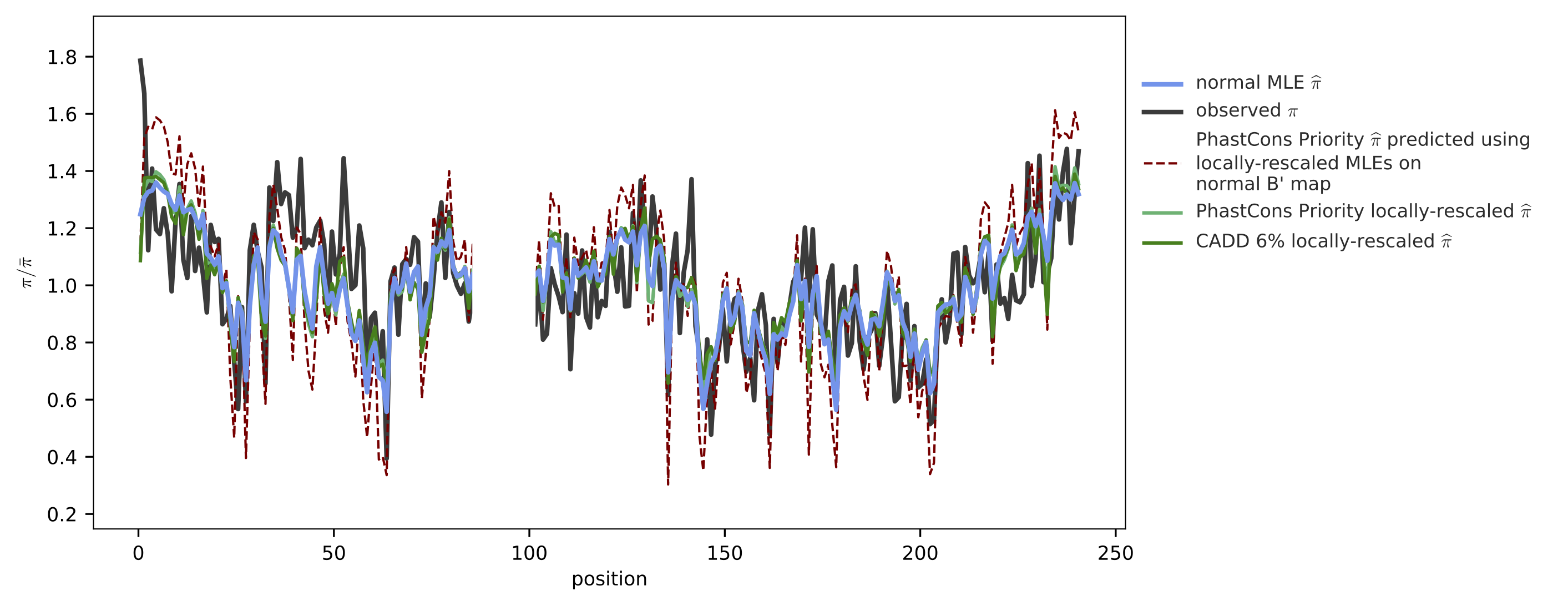

Supplement: S9 Fig — The observed data is the dark gray line, and the normal MLE for the PhastCons Priority model is the blue line. The locally rescaled predictions are the green line. The dashed red line are the prediction using the standard B’ map (without local rescaling) and the maximum likelihood estimates from the locally rescaled fits. The large discrepancy in this shows that estimates are highly dependent on the B’ map. (TIF) [file pgen.1011144.s010.tif]

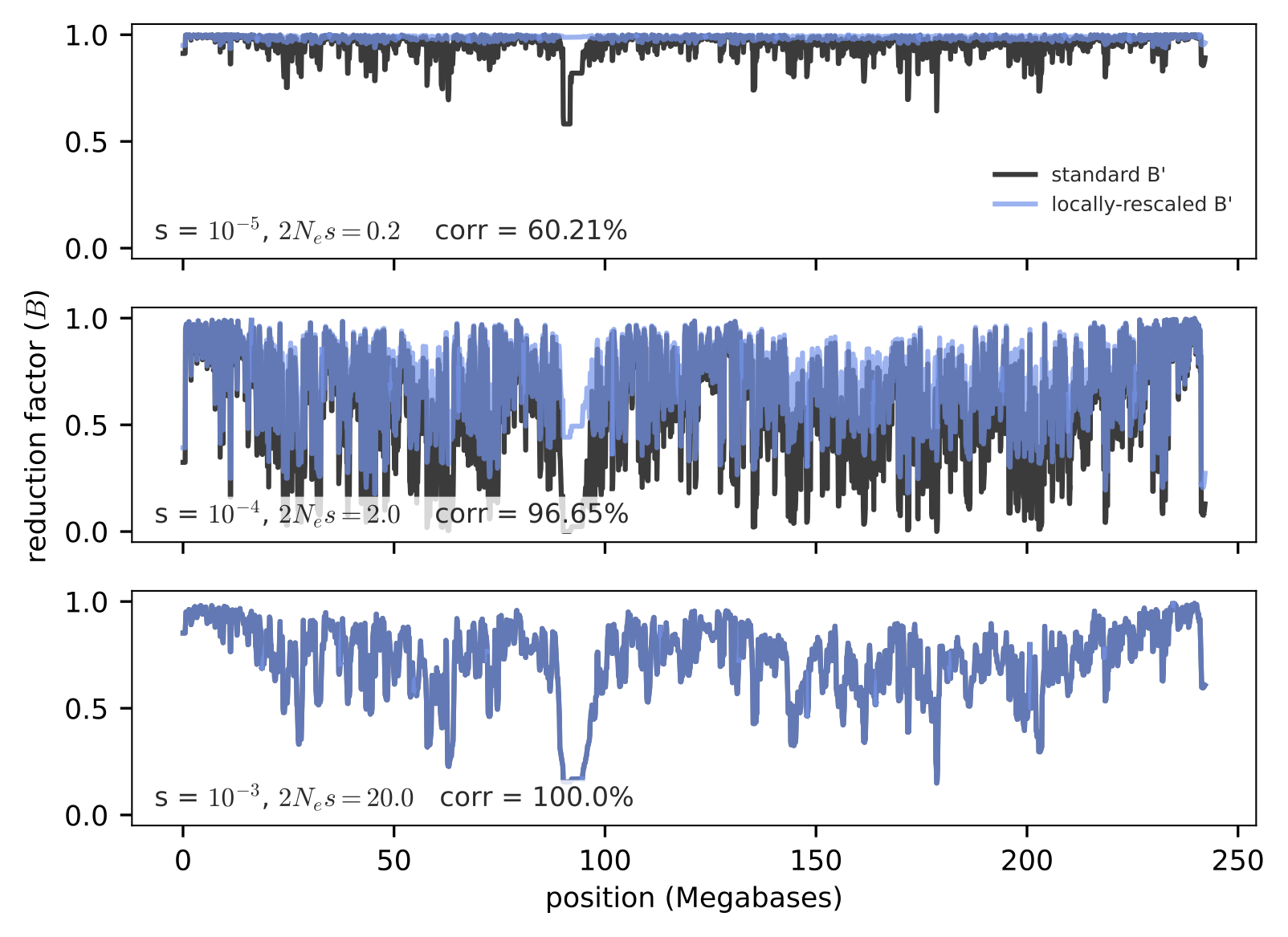

Supplement: S10 Fig — For s = 10−5 (2Nes = 0.2), locally rescaling alters the predicted reduction so that it is essentially insignificant (B ≈ 1). For mid-strength selection s = 10−4 (2Nes = 2), there is only a very slight difference between standard and locally rescaled B’ maps. Finally, for strong selection s = 10−3 (2Nes = 20), local rescaling does not change the B’ maps, as expected. (TIF) [file pgen.1011144.s011.tif]

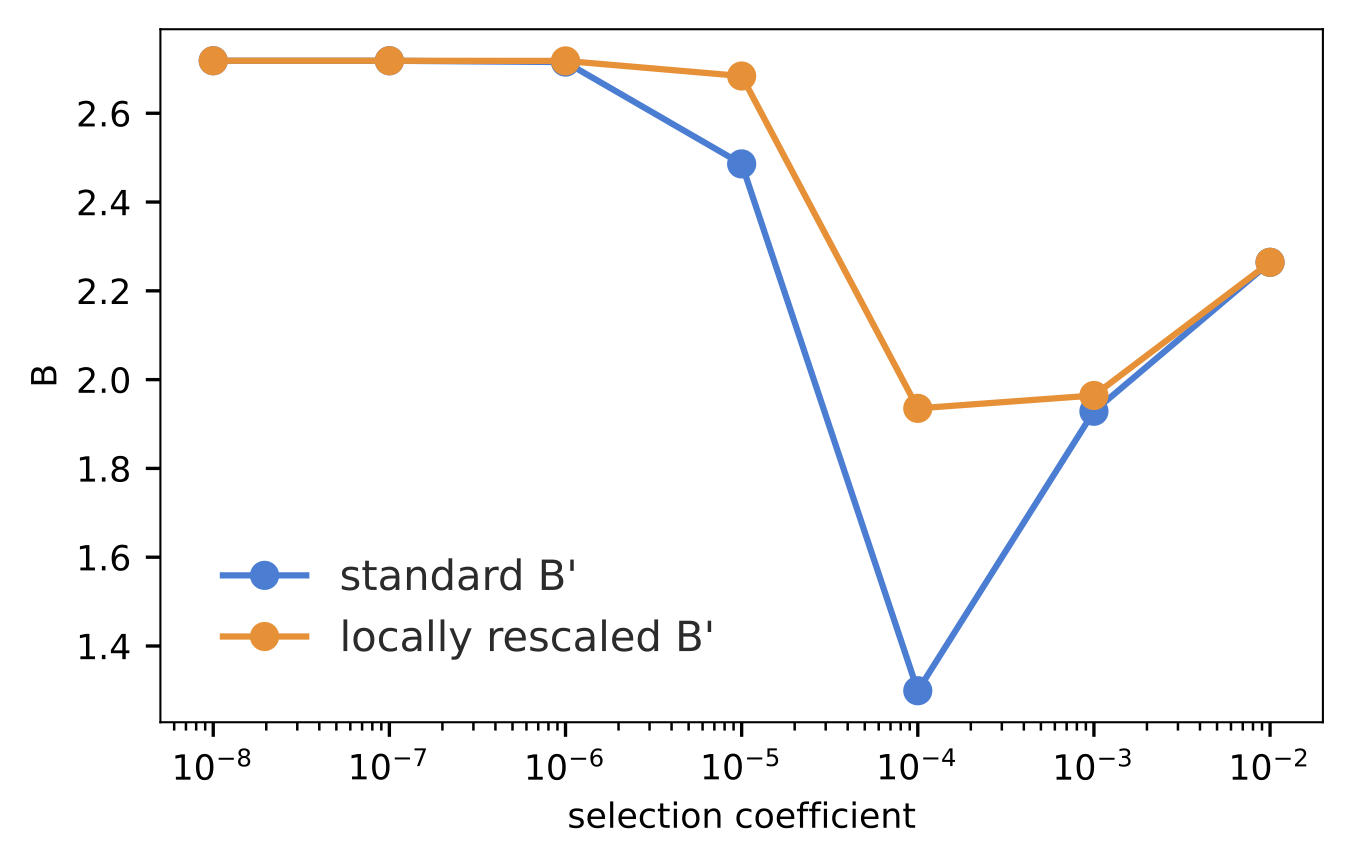

Supplement: S11 Fig — This indicates that locally rescaling the B’ maps only in practice changes how deep the “U” is. (TIF) [file pgen.1011144.s012.tif]
