# Supplementary material for: A quantitative genetic model of background selection in humans
Source: PLoS Genet. 2024 Mar 20;20(3):e1011144. doi: 10.1371/journal.pgen.1011144 (PMC10984650; doi:10.1371/journal.pgen.1011144)
Supplement: Attachment — Submitted filename: PLoS Genetics Response.pdf [file pgen.1011144.s013.pdf]

We thank the reviewers and AE for their very helpful suggestions to our manuscript. We have incorporated their suggestions into the manuscript, generated seven new supplementary figures to address their helpful points. We reply to each of their points below; to make it clear, we have included the original comments in blue and our replies are in black font.

Reviewer #1: Statement: I read an earlier version of this manuscript and provided the authors with some comments, as noted in the Acknowledgments section. However, I am in a good position to give an unbiased evaluation, since there has been no other previous collaboration between us.
E. Santiago

Here, the authors apply the model of Santiago and Caballero (2016) to explain the observed diversity and the divergence rate in humans, assuming that most selective forces shaping variation are deleterious. **While this model takes into account the development of negative LD between selective variants (i.e., the Hill-Robertson interference), which leads to a reduction of the additive variance for fitness (i.e. to a reduction of the effectiveness of selection),** the classical background selection model (BGS) does not predict this effect and consequently ignores the increased rate of fixation of deleterious variants when the product of the Ne and the selective effect (s) is small. This aspect is of particular interest in the human lineage, which has experienced long periods of bottlenecks that have led to reduced nucleotide diversity and gene loss, the latter presumably due to increased accumulation of mutations in non-essential genes. The authors have done a considerable amount of good work, correctly applying the theory and comparing the predictions with those of the BGS model. Simulations show the superiority of the SC16 theory in predicting all aspects of diversity and divergence, especially when selection is weak. **However, some deviations from observations remain, particularly in the prediction of mutation rates.**

I have found that the quantitative predictions follow the basics of the theory, but disagree on some interpretations of the results. **The most important is due to the misconception that the SC16 model does not take into account for the Hill-Robertson interference, which however is believed to be accounted for by simply accumulating the effects of segments along the chromosomes.** I hope that the following comments help to improve the manuscript:

We thank the reviewer for their careful attention they have paid to our manuscript and have tried to make all of the suggested changes.

L64 – I agree that the classical BGS model does not take the Hill-Robertson interference into account at all. But the sentence suggests that this is an issue related to the division of the genome into segments. It says that BGS assumes no interference between segments… but says nothing about within segments.

We agree with this reviewer that this was unclear, and have reworked the mention of segments. We have changed this to read, “[...] Finally, the classic BGS model assumes that the selective dynamics at one site is not impacted by selection at other positions, i.e. no selective or “Hill--Robertson" interference [...]”.

A second point regarding this sentence is that I have not been able to find out how long the segments are, given either by a fixed number of Mbs, or by an interval, or by a criterium. There is also little information about recombination rates, which are as important as the selection intensities for background selection models and can vary across the genome.

With respect to the segments mentioned in our initial submission – these are dependent on the particular annotation model used (now introduced on p. 12). Each annotation model is defined as a set of genomic ranges (start and end positions) for each chromosome (e.g. the coordinates of all coding sequences, or the positions of conserved sites according to PhastCons). Since we do not know *a priori* which annotation model is closest to reality, we have performed model comparison of different annotation models, each containing different segments. We agree with this reviewer that how this was initially presented was unclear, and we have clarified this on lines 128-132, “summation in Equation \eqref{eq:polygenic_Ne} over non-overlapping contiguous,conserved segments $i \in \{1, 2, \ldots, S\}$ (e.g. exons, splice sites, etc.)”. We have also added more detail about the segments on lines 329-331, in the section about annotation models.

L180 – Several citations are given regarding the opinion that determining the rate of the Muller´s ratchet in asexual species is a difficult problem. The paper by Santiago and Caballero (2016) should also be cited here, as their Figure 4 shows the predictions of the ratchet rate by the SC16 model along with the predictions by the other methods. In general, SC16 predicts better than the other methods by simply using the same set of equations used in the manuscript.

We agree, and have added a citation to Santiago and Caballero (2016) in this section.

At equilibrium, there is on average one fixation per click of the ratchet (Higgs and Woodcock 1995), so predicting the fixation rate, which is implicit in SC16 method, is the same as predicting the ratchet rate. The equations in the SC16 method also connect the fixation rate (substitution rate) with the reduction of the additive variance (i.e. reduction of the effectiveness of selection due to negative LD between selected sites in the so-called the Hill-Robertson interference), making it clear that the Muller´s ratchet is the consequence of the Hill-Robertson interference in asexual species, as pointed out by Felsenstein (1974).

We agree that the relationship between average substitution rates and the ratchet rate was not clear in this version of the paper. We have clarified this on lines 186-189.

L267-275 – Although it is not clearly explained how the local re-scaling of the B maps is done, this paragraph suggests that the focal segment is considered twice, or its weight is somehow

increased over the true contribution. Perhaps a simple simulation could help to assess the excess of effect of the re-scaling.

We have done the suggested simulation — in Figure 1E the yellow line shows that local rescaling brings the predicted reduction levels B closer to the simulated values. Ultimately we believe this effect is minor and does not affect our conclusions

Also at the beginning of the paragraph, it is said that the interference between segments is not considered in the set of equations of the SC16 model. Selected sites (or segments) accumulate their effects on the linked focal site (or segment) as shown by the sums and integrals in equations (3) and (4) and other equations in the Appendix. This accumulation is also considered in the classical BGS model. Interference means LD disequilibrium between selected sites, which is also considered in the SC16 model but not in the classical BGS model. Solving the set of equations for a single segment, i.e. ignoring the existence of the rest of the genome, leads to the overprediction of Ne because there are no linked segments to accumulate their effects on the focal segment. In addition, the negative LD with selected sites on other segments, if exists, disappears but this probably contributes little to the variance because most LD occurs between tightly linked sites, most of which would be expected to be found within the same segment, unless the segment were tiny.

We thank this reviewer for clarifying this — we have now significantly edited this section of the manuscript. We believe our prior language was unclear as to what local rescaling is doing. Essentially, the motivation is that it is computationally impossible to numerically solve the Santiago and Caballero (2016) equations (hereafter, SC16 equations) within each iteration of our likelihood framework, in a way that considers all segments simultaneously. The local rescaling approach is an approximation to this that solves the SC16 equations for each segment, but the background N in their equation is given by B(x) N, where B(x) has been estimated from the initial round of model fitting. This is similar to an iterative approach to fitting (which Reviewer #3 suggested too, but given that huge computational barriers to genome-wide likelihood inference, this was not possible at this scale at the time this manuscript was written).

L-282 of the main text and L-343 of the Appendix – It is stated that V_a in eq.(5) [eq.(43) in the Appendix] is the additive “genic variance”. I understand that this means the sum of the contributions of all the selected loci as if they were independent (uncorrelated), i.e. breaking all the associations between loci and adding their contributions. If so, then this concept in the manuscript is wrong according to the SC16 model. Fisher’s fundamental theorem states that the rate of increase of fitness per generation is equal to the additive variance for fitness. This applies exactly in selection models without dominance because it is a definition of the additive genetic variance (Li 1967). Of course it refers only to the variance that is visible to natural selection, so the variance hidden behind negative linkage disequilibrium is not accounted for in V_a. Fisher’s equation is in the center of the SC16 model, which means that the variance hidden behind LD is ignored when predicting the rate of fixation and the effective population size. However, the hidden variability contributes to the nucleotide diversity in an amount that is

ignored by the model, leading to an overprediction of the mutation rate when trying to explain the observed diversity in the range of parameters where the negative LD occurs, mainly when Nes<5 approx. , as demonstrated by solving the set of equations of the SC16 model and also shown by simulations in Figure 1D (top).

We agree with this reviewer, and have removed this section. We have kept the LD plot in Figure 1D since we believe it will be helpful to readers' intuition to see the LD build up.

L284 – Is D a covariance of gene frequencies? It is probably not necessary to include this term in the main text, since it is only used here.

Yes, this is correct — however, as mentioned above, we agree with this reviewer that we were initially wrong, and the SC16 model is modeling total variation, not just genic variation, and consequently we have removed this section.

L297, L305, L321, L357, L494 – Some references to figures or parts of figures are incorrect.

Thank you, we have fixed these issues.

L638 , L676– Certainly, local rescaling, which is a tactic to account for the cumulative contribution of selected sites located beyond the limits of the segment, has little to do with Hill-Robertson interference. L690 , L698 – Of course, like neutral alleles any selected allele will experience the increase in drift caused by selection acting on linked sites. However, if selection is strong (i.e. Nes>>1), this will not significantly affect the evolution of their frequencies. On the contrary, weakly selected alleles may become fixed as a consequence of the Hill-Robertson interference.

Thanks, we have reworked this section on lines 628-630 of the PDF.

L729-740 – Trying to explain the overestimation of mutations rates, several factors not considered in the model are simply mentioned without discussion of how they might affect the estimates. Two of these seem obvious. The first, but probably less important, is that the model predicts well the observed additive fitness variance, but ignores the hidden variance, which however contributes to genomic diversity. The second is that many deleterious mutations are recessive. While the fixation probabilities of these mutations are not much higher than the fixation probabilities of additive mutations with similar effects, the contribution of recessive mutations to diversity is expected to be higher than that of additive mutations because they fluctuate at higher frequencies and for longer times in the population.

We have added further mention of the issue that deleterious mutations may be recessive in the Discussion (we also note that we did forward simulations that show this, i.e. Supplementary Figure 14).

Eq.(32) of the Appendix – L was not previously defined in the Appendix. Assuming that L is the

number of sites along the chromosome, the sum must be from 1 to L/2 since the focal site is in the center of the chromosome. Also, in the integral, the term “(r)” must be a subscript.

Thanks for catching this. We have corrected this issue.

L327 in Appendix - The "r" is used to represent both the recombination rate and the rate of accumulation of deleterious mutations along the main text and the Appendix, which can lead to confusion.

Again thank you for spotting this. We have fixed this issue.

Reviewer #2: The manuscript by Buffalo & Kern models linked selection across the human genomes using theory from quantitative genetics. Specifically, existing theory (e.g. Stantiago & Caballero 1995; 1998; 2016) describes how selection will influence Ne at a neutral locus linked to a deleterious locus by accounting for the increased variance in reproductive success due to the selective effects. In some parts of the parameter space, this model simplifies to the other background selection theory. However, in the case of weaker selection coefficients, classical background selection theory does not work well because it does not account for the possibility of deleterious mutations drifting upwards in frequency. The authors show how the present theory discussed here provides a more accurate model of linked selection in that part of the parameter space. The authors implement this theory into an inference framework to quantify background selection across the human genome. They obtain estimates of a number of key parameters—such as the deleterious mutation rate, and the distribution of fitness effects (DFE).

Overall, I enjoyed this paper. There are a lot of parts to this manuscript and I think it could be a significant contribution. Key strengths include a more accurate model of background selection via a clever application of previous theory from Santiago and Caballero, a sophisticated inference framework to estimate the parameters from the models, comparing the performance of the new and old models of background selection using simulations, and the analysis of human data.

Thanks for the supportive comments!

However, I have a number of questions that I would like to see addressed before the manuscript is considered further for publication.

Major comments:

1) My biggest concern is that some of the estimates of the DFE do not make much sense and are less consistent with previous studies. More specifically, the authors infer that 75% of

noncoding phastCons sites have s around 0.01. Other versions of the results in Figure 4 have qualitatively similar patterns for other annotations of noncoding mutations. However, the estimates in Figure 4 show that coding regions have far fewer mutations under strong selection. While I appreciate that the phastCons regions are indeed likely enriched for deleterious mutations, I'm not sure they are more deleterious than amino acid changing mutations. I also appreciated that synonymous mutations are in the CDS category and likely have very small s. Even still, I don't think this is enough to fully account for the CDS DFE being less deleterious than the noncoding DFE. These results also appear inconsistent with previous work.

Indeed, previous work inferred DFEs on human mouse conserved regions that were less deleterious than those for nonsynonymous mutations (Torgerson et al. 2009 PLoS Genetics). More recently, Dukler et al. (2022), taking a different inference approach, also found that CDS mutations experienced a greater reduction in variation than various noncoding annotations. Thus, I'm not sure the DFEs inferred here are plausible. Indeed, the authors seem to imply that their parameter estimates have considerable uncertainty. When they do the local rescaling to correct for interference, the DFE parameters change a lot. I'm not sure what can or should be done about this. I think this issue could stem from some sort of model misspecification (e.g. not modeling structural variants/transposons/STRs that have some fitness effect that gets sopped up in the other annotations; some of the sites classified as "neutral" actually being deleterious, etc). Maybe greater discussion of this topic and less literal interpretation of the DFE parameters would be in order?

Thank you for bringing attention to our DFE estimates— we think other readers would have similar concerns. There are a number of things to reply to here:

1. Yes, while amino acid mutations may be under stronger constraint than many noncoding portions of the genome, our CDS estimates come from a combination of nonsynonymous and synonymous sites (as the reviewer acknowledges). Specifically, about 70% of single basepair mutations in mammalian coding sequences produce a change in an amino acid (Kryikov et al. 2007, see also Charlesworth and Charlesworth 2010, p. 255). Thus ~30% of mutations in coding sequences are not expected to induce an amino acid change, and are effectively neutral. Our DFE estimates from noncoding regions, by contrast, come from PhastCons regions that were ascertained by searching for highly constrained sequences in distantly related vertebrates (Siepel et al., 2005). PhastCons elements do not cover all coding basepairs, but instead only include about 66% of CDS (consistent with synonymous sites being unconstrained and thus excluded). So, suppose that ~80% of all non-coding PhastCons mutations have about s = 0.01 (as in Figure 4B). Now, if these were coding sites, 30% of the basepairs in these regions would have effectively neutral mutations because they'd be synonymous. This would leave about $0.8(1-0.3) \approx 56\%$ of mutations at s = 0.01, which is close to what we see as our estimate of the DFE estimates for coding sequences in CEU/CHB in Figure 4B. We do note that YRI has slightly different behavior in this CDS-priority model, but this model fits the most poorly out of all models (but we highlight it because its feature categories are the most interpretable). We take this as decent evidence that our DFE estimates are

not wildly different from expectations. We have added text pointing this out in the relevant Results section.

2. Our results do contrast with Dukler et. al's results on 'ultraconservation', in which they reported conserved noncoding sequences to be under less constraint than CDS. As Dukler et al. point out, their DFE estimates for CDS indicate much stronger selection than previous estimates based on the site frequency spectrum (as ours essentially are). Dukler et al, because they are using aggregated, super deep samples from GNOMAD, may be able to capture a stronger signal of selection that we are capable of detecting in 1000 Genomes data. We had added text pointing out this difference in the Discussion.

As an aside, I do not think that having the "best" estimates of the DFE parameters is a requirement for the present paper to be important. The B' value map along the genome is an important enough result, in my view.

We agree. We indicate in the discussion that (line 720) that predicted diversity differs little between DFE and mutation rate estimates, suggesting the information about these parameters in the linked selection signal is somewhat limited. However, we find parameter estimates are relatively stable across different reference populations, suggesting these estimates are not being driven by noise.

3) As the authors acknowledge, recently Murphy et al. (2022) also presented a model of background selection across the human genome. Their model has some similarities and differences compared to the model used in the present manuscript. While the authors compare the models to see which one has a higher R^2 with the empirical data, I think a more in-depth comparison would strengthen the present manuscript. Specifically, how correlated are the sets of B-values across the genome? Are there specific regions that depart more than others? Examination of such regions might reveal some significant insights.

We have included a new Supplementary Figure (Supp. Fig. 26, p. 62) showing the ratio of our classic B and B' for the CADD 6% model. The Murphy et al. data is on a different reference genome and recombination map, but since it's using the same theory, it's likely very similar to our classic B. Overall the correspondence in predicted B and B' maps is very close (R2 of 99.99%), as expected given that the CADD 6% sites are very likely under strong constraint, and in this domain, the models are identical. We have added mention of this comparison in the main text on lines 395-396. We note that there are some regions that do differ as apparent from our added Supplementary Figure. These regions that differ in B and B' are just due to numeric instability in centromeric regions (due to recombination rate being zero). These regions are masked in our data analyses, so have no effect on results.

4) Line 449: The authors apply their approach to infer parameters across several continental

ancestry groups. Overall, they find that the inferred DFEs are fairly similar across populations (Figure 4). I agree with the authors that this is an encouraging sign that their inference is not too confounded by demography. I also applaud the authors for looking at how demography influences selection. However, as human populations share a recent common ancestor, I would not expect the DFEs of s to differ too much between populations. Indeed, recent work suggested the DFEs are quite similar (Huang et al. 2021 MBE). What is different between populations is the demographic history. Thus, this means that the patterns of variation may be different across different populations. I believe this would come through in the B' values that are inferred for each population. How concordant are they across populations? Regions of the genome with different B-values in certain populations could be illustrative of interesting biology (maybe local adaptation, even). I think some additional investigation on this is warranted.

We agree that it could be interesting how the B' values differ across populations. We show in Figure 1A how predicted diversity is very similar across the different populations (represented by the different colors). Since predicted diversity at position x is $\pi(x) = \pi0\ B'(x)$, and we have mean-scaled the y-axis, the close correspondence in this figure shows how the B' maps are very similar across species. Still, we agree that looking at scatter plot comparisons and the correlations between populations could be of interest to readers, so we have included a new supplementary figure (Supp. Figure 27) and mentioned this on line 548 of the main manuscript. For now, we hold off on looking at and interpreting these outliers, but the data is available for others to do so.

5) The authors would greatly strengthen the impact of their work if they made a nicely curated set of B' values along the genome easily available to readers. I think this would be an invaluable resource, especially since the original McVicker B-values are still widely-used.

This is a nice idea. We have added a file of the B' map, along with average recombination rate, pairwise diversity, and some other summary statistics for each population to the Data Dryad repository.

6) Much of the paper focuses on the "sparse track" and the "full track". These terms should, at least briefly, be explained clearly in the main text before Figure 2 or the first time they're used. They appear over and over again, so a reviewer (or reader) might get lost.

Sorry for this initial deficit. We have added a definition for each of these at the beginning of the annotation model section (lines 346-348).

Minor comments:

1) Line 42: McVicker et al. (2009) actually fit their model to divergence data among primates. They then looked to see how correlated it is with patterns of diversity. Please consider rephrasing.

We have changed the phrasing here to address this issue.

2) Line 203: The switch to talking about the deleterious substitution rate appears a bit abrupt. It wasn't immediately clear to me why we're making the switch to talk about substitutions. Is this to get an extra piece of information from the data (ie substitutions) so that we can better infer parameters (as implied in equations 6 and 7)?

We agree with this and the other reviewers that this was unclear, so we have changed mention of the ratchet to substitution rates. This is a more accurate description of what is going on.

3) Line 237: I believe Figure 11C should be Figure 1C.

Thank you, we have made this change.

4) Line 282-286: I had a hard time following this section here. How does the negative LD lead to the error?

Following a correction from another reviewer, we have removed this section.

5) Line 356: Is it that the linked selection effect actually matters most at the Mb scale? Or is it that patterns of polymorphism are so noisy at smaller scales, that it's impossible to detect? I think these are different things, and some clarification here would help.

It is a ratio of both — at the megabase scale the noise from the coalescent process and mutations is diminished relative to the linked selection signal by averaging at these large scales. This is related to the R_coal^2 that we develop, discussed in the main text and Supplementary Section 3.8 (p. 31). We agree that this is likely helpful to readers to point out, and have added a sentence about this point on lines 366-368.

6) Line 468: I believe you mean Figure 4A instead of Figure 3A.

We have fixed this issue.

7) How do the B' value maps correlate with recombination rates?

This is a great question — B' (and B) positively correlates very strongly with recombination rate (this can be seen approximately by classic theory, where B = exp(-U/R) [e.g. from Hudson and Kaplan, 1995] dB/dR = exp(-U/R) U/R^2 which is strictly positive). We thought this would make a great supplementary figure, so we have added it (Supplementary Figure 22, p. 60). We have also added additional relevant diagnostic supplementary figures inspired by this suggestion: residuals plotted against recombination rate (Supplementary Figure 21), average pairwise diversity by recombination rate (Supplementary Figure 23), average pairwise diversity by fraction of window overlapping CADD 6% features (Supplementary Figure 24). We have also added a short mention of some of these figures (lines 570-573).

Reviewer #3

Reviewer #3: In this study, Buffalo & Kern investigate how purifying selection shapes the patterns of genetic diversity along the human genome. To do so, they extend an approach initially developed by McVicker et al (PLoS Genet 2009), and later generalized by Murphy et al (eLife 2022) & others, which attempts to fit the megabase-scale variation in pairwise neutral diversity across the genome using a "background selection" model based on the locations of conserved genomic regions. The key difference in this manuscript, compared to previous work, is that they attempt to properly account for the effects of weak selection. This is a longstanding issue in the background selection literature, but it has been neglected in all previous studies that attempt to fit the background selection model to the human genome.

Buffalo & Kern overcome this issue by employing a theoretical approximation developed by Santiago & Caballero (Genetics 2016), which has previously been shown to capture some of the effects of weak selection on pairwise neutral diversity. The authors use this approximation to develop a genome-wide model for the local reduction in pairwise diversity, B'(x), as a function of the recombination map, and the distribution of deleterious fitness effects in different classes of conserved regions. They propose an efficient approach for fitting this model to observed pairwise diversity data from humans, which allows them to obtain predictions for B'(x), as well as estimates for the corresponding DFEs.

Overall, I think this is a nice study that has the potential to significantly advance our understanding of linked selection in the human genome. While the problems created by weak selection have been recognized for some time, our inability to incorporate them into empirical analyses like this one hampered our ability to determine whether simple models of background selection are sufficient to recapitulate the observed data. I have a couple of technical comments, as well as suggestions for improving the clarity and accuracy of the exposition.

We thank this reviewer for these nice comments and very helpful feedback overall.

Major points:

(1) I think it would be helpful to acknowledge more of the limitations of the Robertson/SC16 approximation when it is introduced in the main text (starting from Eq. 1). For example, previous work has shown that background selection *can't* be summarized by a rescaled effective population size (see. e.g. Good et al PLoS Genet 2014, Cvijovic et al Genetics 2018). But a naïve reading of the exposition in the Theory section seems to imply that it *can*, and that the main problem is how to pick the right value.
I think the issue stems from the authors' proof of Eq. 1 in SI Section 1. In a more detailed calculation of the asexual case, Cvijovic et al (Genetics 2018) showed that this genetic drift mapping only holds on timescales longer much longer than the autocorrelation timescale. This can be useful for some observables (e.g. the pairwise neutral heterozygosity) but not for others (e.g. site frequency spectra or substitution rates) that depend on dynamics on shorter timescales. For example, the functional form of the substitution rate in Eq. 7 is not correct, as

studies like Neher and Shraiman (Genetics 2012), Good et al (PLoS Genet 2014), and Melissa et al (Genetics 2022) have shown.

We agree with this reviewer, and have made several changes to emphasize the limitations of the background selection model. First, on lines 94-99 we have included these citations and made clearer to readers that the Santiago and Caballero model is an approximation, and may not give accurate results across all regions of parameter space. We have included additional text on line 222 about this, citing these papers.

(2) I was a bit confused about the definition of a "segment", first mentioned on line 130. How long are these and how is that length scale chosen? Is it related to the "linkage block" concept described in Good et al (PLoS Genet 2014)? How is it related to the concept of a "window" as used for data fitting?

Segments are of arbitrary size; they are set by the particular annotation model specified. In particular, each annotation model takes in a BED file of putatively conserved range, which can vary in length. We have added text to clarify this to the reader on lines 262-263. By contrast, a window defines the scale at which we average diversity over and fit it in our inference approach.

On one hand, it sounds like the segments must be longer than a single site (otherwise Eq. 6 and 7 would return Nf very close to N). But on the other hand, the definition in Eq. 7 makes it seem like there is a single mutation rate and selection coefficient (rather than the full DFE described elsewhere). Later on in the Methods section (Eq. 14), it seems as if different segments are also used to capture different parts of the DFE. How do we reconcile these seemingly conflicting approximations? A bit more discussion of this issue in the text would be helpful.

This is correct — Nf is very close to N for a single site, such that $B < 1$ but B is close to 1. This reflects the fact that a single site has very little impact on a neighbor's diversity through linked selection, but cumulatively (through the product) the effect does contribute.

We note that in equations 5-7 we are just considering a single segment, to try to simplify the exposition to readers. We have added sentences clarifying this earlier in this section, as well as indicating at the end of the theory section that we later extend these equations to handle (1) a distribution of selection coefficients and (2) a multiple feature classes.

The same approximation is used in both equations — essentially, our approach computes the SC16 equations for each segment for a grid of mutation rate and selection coefficients. Since varying the mutation rate is the same as setting a genome-wide mutation parameter μ and varying its weights per feature class x selection coefficient grid element, these work out to be the same. Each segment can be assigned to one feature class and a distribution of selection coefficients. We note that this approach to estimating the DFE is used in other works (Eyalshiv et al. 2016 and Murphy et al. 2022).

(3) On a related point, I was confused about the transition from Eq. 2 to Eq. 6 – why does the sum over segments disappear in going from Eq. 2 to Eq. 6?

We omit the indices for clarity — throughout this section we tried to balance ease of explanation for a non-mathematical audience without losing too much rigor. We have clarified this a bit.

This could be related to the “locally rescaled B’(x)” mentioned later in the paper, which I was also somewhat confused about. Isn’t the locally rescaled version the “correct” version of Eq. 2? It seems like the most straightforward application of the SC16 model would result in an implicit equation for B’(x) of the form

B’(i) = exp[- sum_j mu_j s_j (1- 4N B’(j) s_j / (exp(4N B’(j)) – 1)) * Q_ij^2 / 2]

with Eq. 6 and the “locally rescaled B’(x)” corresponding to different approximations that drop certain terms in the sum. Is it possible to just solve this equation directly, e.g., by converting to an iterative formula,

B’(i,n+1) = exp[- sum_j mu_j s_j (1- 4N B’(j,n) s_j / (exp(4N B’(j,n)) – 1)) * Q_ij^2 / 2]

and iterating to convergence starting from B’(i,0) = 1? If so, this might be easier to motivate than the current version. Alternatively, the functional form of Q_ij is such that we expect very distant segments to contribute little to the sum. One could use this intuition to restrict the sum to some maximum distance, so that full quadratic complexity isn’t needed to calculate B’(i). Depending on the length scale chosen for the segments, it may be that Eq. 6 is doing exactly this. But the differences between the initial B’(x) and the “locally rescaled B’(x)” suggests that the default length scale is neglecting some important contributions in the sum.

This is a great idea — actually, it is related to future planned work. However, this approach was computationally infeasible for human-scale genomic data (especially considering the density of some annotation models, like the CADD 6% one), which is why we opted for the local rescaling approach (this is in essence like one iteration). We also felt that such an implicit form solved iteratively may appear “circular”, hence our work to approach this as an optional alternative model.

We have experimented with reducing the sum to a maximum distance, but this leads to erroneous results (we agree in principle it should work, but even the very weak reductions from distant locations do have a cumulative effect).

(4) The authors return to this issue a bit on line 598, but it felt somewhat strange to read it down there (where it is presented almost as an empirical observation) since it seems to follow directly from how their theory is set up. I also don’t understand the argument on line 616 that one should exclude the segment’s own contribution to B(x). Isn’t that exactly what is included in Eqs. 6 and 7?

This reviewer is correct that the equations include the focal segment's contributions to the reduction. Our concern is that since the B(x) map includes the focal segment, and then we recalculate the SC16 equation models again (at each segment) using that B(x), that the tiny impact a focal segment adds to B(x) is included twice. We have clarified this issue in the text on lines 646-648. We note though that we have done robustness checks (mentioned in that section) that indicate this is not the cause of too much discrepancy. Another way to think about this is that this is a problem that emerges only from our local rescaling approach; the full iterative approach like this reviewer suggested (that is currently computationally infeasible) would not have this issue.

(5) I was confused by the discussion of "genic" vs "genetic" variance on lines 276-288. Does "genic" refer to the entire segment? Or for the contribution of a single site? Conversely, does "genetic" refer to the entire genome? Or to the entire segment? Some additional clarity here could be helpful, since it relates to the point below:

We have removed this section, since Reviewer #1 has pointed out that the SC16 does not model the genic variance (as we mistakenly thought), but rather the additive genetic variance. But, to address this reviewer's question, in theory additive genetic variance should include the whole genome theoretically.

(6) I could not find the derivation in the SI for the claim on line 286 that "according to theory, the reductions in diversity should be determined by levels of additive genetic fitness variance that include the contribution of LD". If I understand the SI derivation correctly, it assumes that different segments are in linkage equilibrium with each other (similar to the "linkage block" ansatz from Good et al PLoS Genet 2014), so it actually refers to the "genic" variance in the sense that it neglects LD between segments (i.e. if "genic" is defined at the segment level).

We have removed this section (as mentioned above). To address this reviewer, we currently think it is uncertain how far the SC16 model considers i.e. whether just in a "linkage block" or LD more broadly across the genome. However, we believe it is the latter, because one can imagine that the SC16 model is much like the Robertson (1961) model, and applies even to unlinked sites in the genome.

Conversely, the "Fisher's fundamental theorem" in Eq. 5 applies for the full genetic variance across all segments (see Eq. C.4 in Good & Desai TPB 2013 for a proof). I was therefore confused about the claim on line 282 that "the equilibrium fitness variance modeled by the SC16 theory is predominantly the additive genic fitness variance". It would help if the authors could clarify which aspects of the SC16 theory they are referring to here.

We thank this reviewer and Reviewer #1 for point this out — we are now in agreement with both that the SC16 model is modeling the additive *genetic* fitness variance and not just the additive genic variance. We have corrected throughout that we are not referring to the additive genic variance.

(e.g. perhaps the authors are referring to the fact that the reason that Eq. 7 is wrong because it is neglecting LD between mutations? In that case I would still argue that it's not an accurate model of the genic variance either…)

This is an interesting point, and we feel that more future research is needed here. Since the SC16 model is based on Fisher's Fundamental Theorem, it should in theory be including the LD between mutations. However, why that is is unclear.

(7) In the main text, the authors talk about inferring a full DFE (e.g. line 290), but in practice they use a discretized version with weight at a fixed # of selection coefficients. I think that's reasonable, but it would be helpful to mention this approximation in the main text, and how the errors it introduces might influence the biological conclusions. E.g. if the true DFE is "off grid" (or a continuous distribution like a Gamma distribution), what kind of errors does it create if these data are fit to the logarithmically-spaced grid that the authors employ here? I'm particularly curious about the interpretation of the inferred parameters, given the later discussion about whether the inferred mutation rate is too high or not. Could model misspecification in the DFE be contributing to this issue?

We agree that model misspecification could be an issue. We used this form for the DFE following previous work (Elyashiv et al. 2016, Murphy et al. 2022). We agree that future approaches should explore whether this logarithmically-spaced grid is appropriate and possible fit alternatives and do model comparison (we added mention of this to line 775 in the discussion). We note too that we had similar concerns about "off grid" issues; this is what motivated us to also go beyond these previous studies, and re-run all of our main model fits on an expanded "strong selection" grid that goes up to 0.1 (lines 451 onwards). We find that there is a degree of non-identifiability here; intuitively what we think is happening is that strongly deleterious mutations are selected away so quickly, they can be difficult to differentiate from a reduced background diversity parameter, π_0 (see Supplementary Figure 11).

(8) I was confused about the use of the term "possible selective interference" in the section title on line 593. I would have thought that this entire paper was looking at the signature of selective interference.. could the authors define what they mean here more explicitly?

We have added several sentences discussing selective interference (in talking with colleagues, it seems many people in the field have different operating definitions). We agree with this reviewer's interpretation, i.e. that when selection creates a lower effective population size (as evident from the inferred B(x) map), this in turn impacts the dynamics at selected sites. One reason we opted to use the local rescaling approach is that it allows us to explicitly separate two issues with the SC16 model. First, it allows us to consider what a segment's contribution to B(x) is when it experiences a background Ne of N, the drift-effective population size. Second, it allows us to check how these results change when the background Ne is B(x)N. We note that (to our knowledge) no previous authors estimating the B map in humans have phrased it in terms of selective interference.

(9) I didn't understand why the molecular clock was necessary on lines 572-576. Doesn't the theory provide a prediction for the relative divergence along the genome, so you could compare these relative values rather than absolute ones?

We agree the mention of the molecular clock was unclear and have removed it. Instead we specify the issue is the heterogeneity in constraint across genes, i.e. some genes may be expressed more or earlier in development, and thus under much more constraint. Our CADD 6% and other models do not allow for and fit this fine-scale heterogeneity, so it acts as an extra layer of noise. This prohibits us from doing the comparison suggested here.

(10) If I understand the description on line 636 correctly, the model misspecification issue in the previous section goes away if you use the locally rescaled B' estimates – is this more evidence that one should be using the iterative re-estimation method from the beginning (as suggested by comment 3 above)? It is worth spending a whole section on the model misspecification issue if it was caused by an approximation that was mainly motivated by computational efficiency?

We agree the iterative approach would have been better, if computationally feasible. However, we do want to keep in the caveats about model misspecification here, because we want readers to know that these limitations of our current approach introduce some uncertainty in our model estimates. We imagine that more refined methods could alter the parameter estimates.

More broadly, this is a general issue I had with presenting several different approximation schemes in the main text, in that it was sometimes difficult to remember which ones had which implications for the inferred parameters / model checks, and which ones I should believe the most.

Through this revision, we have made it more clear to readers that there are several approximations used throughout. The underlying approach of the SC16 model is an approximation that seems to work fairly well in practice (this is why we did extensive simulations — to show this was the case), but it is presently somewhat unclear why it works so well, since it has not been rigorously proven.

(11) Is it possible to do a final forward-time simulation of the authors' "best" parameter estimates (after all caveats have been considered) and use those to generate a final set of B' values? That would eliminate any ambiguity in whether the model approximations are accurate.

Unfortunately, these simulations are not feasible without tremendous time and work. The effective population size estimate commonly cited in humans is Ne=10,000. However, this is based on average diversity levels, which in turn are reduced by BGS. So if average B=0.7, then, the real drift effective population size would be ~33,333. Currently running whole-chromosome forward simulations with selection and realistic recombination maps for population sizes this large is beyond the scope of what we are hoping to do during revision.

Minor:

(12) The statement on lines 117-121 feels a little misleading – as described above, one can only neglect the fitness background of the neutral variant on timescales much longer than the autocorrelation time; this is the same timescale on which the fitness backgrounds equalize in the coalescent approach as well (Nicolaisen and Desai, Genetics 2013). Thus, it's more a benefit of the separation of timescales approximation, rather than the forward-time approach per se.

We are trying to make a different point here, and hopefully have clarified the issue by adding mention of the timescale issue. In particular, we are trying to say that the forward in time models from the neutral allele's perspective are ultimately about figuring out the asymptotic variance, and as such Robertson's model works whether the neutral allele finds itself on a high or low fitness background (we have added this to this section to help clarify this point). Perhaps another way to think about it is that which neutral allele we chose to track is arbitrary, and this imposes some symmetry on the problem when looking forward in time. By contrast, if we sample a lineage and look back in time, the genealogy depends on the absolute fitness; high fitness and low fitness backgrounds are not exchangeable in the same way they are in the Robertson forward model. We have clarified this as such, but welcome suggestions since this is quite a technical point that we want to be accurate:

The benefit of modeling linked selection with Robertson's forward-time model is that the inflation factor under weak selection is invariant with respect to the particular fitness background (i.e. high or low fitness backgrounds are exchangeable in this model) the neutral allele becomes stochastically associated with. By contrast, modeling diversity levels under linked selection backwards in time requires tracking the particular associated fitness backgrounds, as coalescence rates experienced by a lineage are not invariant with respect to their fitness background, i.e. high and low fitness backgrounds are not exchangeable.

(13) Line 205. Neher and Shraiman (Genetics 2012), Good et al (PLoS Genet 2014), and Melissa et al (Genetics 2021) have all proposed methods for calculating the rate of Muller's ratchet in different regimes, including the ones studied here. It would be good to cite these somewhere in this section (perhaps in the discussion of why Eq. 7 breaks down).

We agree, and have added citations to these papers. We welcome feedback on whether this phrasing could be altered to be more technically accurate.

(14) I think the reference to Good and Desai (Genetics 2013) on line 170 should be Good et al (PLoS Genet 2014)?

Thanks for catching this. We have fixed the error.

(15) For clarity, it would be great to use a different looking index (e.g. t) for the sum over generations in Eq. 3.

We have fixed this.

(16) I didn't understand the rationale behind the "stopping condition" in line 266. Wouldn't this always be true if we hadn't simulated enough replicates to begin with? Why do we not simulate more so we can get an absolute estimate of the error? (this seems to be important for the paragraph below this line).

We are not quite sure about the "stopping condition" referred to here. If it is regarding the lower bound $V(B) \approx 2/9n$, our thinking was that the MSE = var + bias^2 where the variance is determined by the variance of simulation replicates around the mean. However, as the number of simulations goes to infinity, there is still the noise of the underlying coalescent processes; Tajima's expression is a rough approximation to this (it is approximate since the BGS coalescent variance is likely lower than the neutral coalescent variance for the same reason discussed in the section on R_coal^2). This is like the "irreducible" error in the MSE decomposition.

(17) There were various places where I didn't understand the indices used in the equations in the Methods section. Eq. 13 is an example (the i is summed over in the exponential, while j is not, but both are arguments of the LHS). If the authors could clarify this notation here and elsewhere that would be helpful.

We thank this reviewer for spotting this important (and embarrassing) mathematical error. The issue is that the inner summation should not be there. This inner summation is over the n_s elements of the selection grid — when calculating the B grids used for fitting, these are done for a pair of mutation rate and selection indices (which are correctly on the LHS). The only summation at this step is the summation across segments, which is the cumulative reduction due to all segments (conditional on the particular mutation rate and selection coefficient grid element). Then, later during fitting, is this inner summation done (with the mutation rates being chosen through the DFE weights, e.g. $m_i = \mu w_{i,j}$)
